# Blood C-reactive protein and white blood cells trajectories and associations with clinical parameters and complications in the acute phase after subarachnoid hemorrhage

**DOI:** 10.1007/s00701-026-07034-8

**Published:** 2026-09-15

**Authors:** Pavlos Vlachogiannis, Anders Hånell, Johan Wikström, Per Enblad, Lars Hillered, Elisabeth Ronne-Engström

**Affiliations:** 1 Department of Medical Sciences, Section of Neurosurgery, Uppsala University, Uppsala, Sweden; 2 Department of Surgical Sciences, Section of Neuroradiology, Uppsala University, Uppsala, Sweden

**Keywords:** Subarachnoid hemorrhage, CRP, White blood cells

## Abstract

**Background:**

Inflammation holds a central role in the pathophysiology of subarachnoid hemorrhage (SAH). Two common non-specific inflammatory markers, C – reactive protein (CRP) and White Blood Cells (WBC), were analyzed in blood of SAH patients and their temporal patterns and associations with bleeding severity and complications were studied.

**Methods:**

The charts of 552 SAH patients admitted between 01.01.2015 – 31.12.2019 were retrospectively analyzed through our clinic’s database. Blood concentrations of CRP (reference range < 5 mg/L) and WBC counts (reference range 3.5–9 × 10^9^/L) were extracted and their temporal trajectories from Day 1 to Day 10 post ictus were recorded. Associations of Day 1 and Day 4 CRP and WBC values with clinical parameters including age, sex, identifiable bleeding source, bleeding severity based on Fisher scale, neurologic status on admission based on Reaction Level Scale-85 (RLS-85) score, occurrence of External Ventricular Drainage (EVD), infections, and Delayed Ischemic Neurologic Deficit (DIND) were examined. Mann Whitney U test was used for univariate analyses in dichotomized groups and General linear/nonlinear models with best subsets of variables based on Akaike’s Information Criterion for multivariate analyses.

**Results:**

CRP peaked on Day 4 and WBC on Day 1. Univariate analyses showed that patients with more severe hemorrhage (i.e., identifiable bleeding source, Fisher 3–4, RLS 4–8, EVD indication) and complications (infections, DIND) had significantly higher CRP and WBC values on Day 1 and Day 4. Multivariate analyses showed that DIND was among the variables included in the three models most likely to be associated with higher Day 4 WBC but not CRP values.

**Conclusions:**

More severe bleeding and occurrence of complications in the early phase of SAH are associated with a more intense inflammatory response. An increase in WBC (rather than CRP) around Day 4 post ictus may be associated with the occurrence of DIND.

## Introduction

The brain pathophysiology of spontaneous subarachnoid hemorrhage (SAH) is a complex multifactorial process that involves several cellular, molecular, and physiological mechanisms [5, 41, 42]. Examples of these mechanisms are endothelial damage, oxidative stress, activation and aggregation of white blood cells and platelets, cell death pathways, activation of inflammatory cascades, impaired ionic homeostasis, and alterations in physiological parameters such as intracranial pressure (ICP), cerebral perfusion pressure (CPP), and cerebral autoregulation [4, 28, 40]. Additional systemic reactions including endocrine changes, cardiovascular stress, infections, and other complications may also occur in the acute phase. The exact interplay between these mechanisms and their temporal evolution is difficult to define and there is a great variability that depends on genetic, environmental, and other factors [7, 39].

The role of neuroinflammation in the occurrence of brain injury following SAH has been demonstrated by numerous clinical and experimental studies [43]. The concepts of Early Brain Injury and Delayed Cerebral Ischemia (DCI)/Delayed Ischemic Neurological Deficit (DIND) are commonly used and inflammatory processes are reported to be present in both [6, 27]. Several inflammatory cells and molecules have been associated with the occurrence of SAH complications and clinical outcome [10, 21, 32, 35].

Blood C—reactive protein (CRP) and White Blood Cells (WBC) are common non-specific markers of inflammation that have been studied in the context of numerous systemic and neurologic diseases including for example atherosclerotic disease, ischemic and hemorrhagic stroke, neurotrauma, and infections [3, 12, 29, 38]. CRP is an acute phase plasma protein produced in the liver in response to increased levels of Interleukin 6 (IL-6), caused by e.g., tissue injury, inflammation, and bacterial infection. CRP is a highly dynamic biomarker with an increased plasma concentration detectable already about 8 h following tissue injury. Likewise, a secondary peak following normalization is useful as an early signal of secondary injury. WBC counts reflect mainly the concentrations of neutrophil granulocytes and lymphocytes, i.e., the two major WBC types. Since the neutrophils account for 50–75% of the WBCs, WBC counts are used to estimate the severity of an inflammatory reaction involving neutrophils. Both CRP and WBC counts are useful for monitoring of the temporal evolution of the inflammatory activity. Elevated CRP and WBC levels in blood and cerebrospinal fluid (CSF), as well as other features such as neutrophil–lymphocyte ratio, have been associated with the occurrence of DCI and poor outcome after SAH [1, 15, 17, 22, 24, 49].

In the present retrospective study, CRP and WBC concentrations were analyzed in a large cohort of patients with varying grades of SAH in the early phase of the disease. The central idea of the study was that the elicited inflammatory response is the result of the presence of blood in the subarachnoid space, regardless of the existence of aneurysm or other identifiable bleeding cause; thus, we chose to analyze aneurysmal SAH together with non-aneurysmal SAH. The aims of the study were to examine if there were specific trajectories over time for these two parameters and how they were associated to the disease course; to determine how the admission values were affected by demographics (i.e., age, sex) or factors reflecting Early Brain Injury, such as amount of blood on initial radiographic examination and neurological condition; and, finally, to determine how the CRP and WBC values on Day 4, i.e., when many complications have occurred, were affected by Early Brain Injury and common SAH complications such as infections and DIND.

## Methods

### Patients

Patients with spontaneous SAH admitted to Uppsala University Hospital’s Neuro-Intensive or Intermediate care unit between 01.01.2015 and 31.12.2019 were enrolled in the study. Data were extracted from the clinic’s administrative vascular database and from reviewing medical records. The diagnosis was established by brain Computerized Tomography (CT) scans that were classified according to the Fisher scale [14]. Lumbar puncture was used to confirm the diagnosis whenever CT scan did not show evidence of subarachnoid blood, but the clinical picture was strongly indicating SAH. All patients underwent CT angiography (CTA) and, when indicated, Digital Subtraction Angiography (DSA) for the identification of the bleeding source. Neurological status on admission was recorded based on the Reaction Level Scale (RLS)−85 score [44]. This scale is comparable with the motor component of Glasgow Coma Scale with a cut-off between conscious (RLS 1 to 3) and unconscious patients (RLS 4 to 8) allowing for comparisons in clearly defined dichotomized clinical groups. Unconscious patients (RLS > 4) were mechanically ventilated and sedated with continuous infusion of propofol and morphine. Neurological status in sedated patients was checked with regular wake-up tests. External Ventricular Drainage (EVD) was inserted in unconscious patients not responding to commands. Rebleeding was confirmed with CT scans when suspected clinically; however, rebleeding occurred in only a few patients (*n* = 12; 2,1%) and this parameter was therefore excluded from further analyses. Infections were confirmed with positive cultures and comprised meningitis, bacteriemia/sepsis, urinary tract infections, clostridium infections, and skin infections. In ventilator-associated pneumonias chest x-ray was used for diagnosis. Delayed Ischemic Neurological Deficit (DIND) was defined in accordance with Vergouwen et al.’s proposal as a focal deficit and/or decrease in the level of consciousness not attributable to aneurysm treatment, new hemorrhage or hydrocephalus, and/or as delayed cerebral ischemia (DCI) seen on CT scan [46]. Patients with DIND were treated with induced hypertension, hypervolemia and hemodilution, if no contraindications existed. Endovascular treatments, such as balloon dilatations of large vessels or intraarterial injections of vasodilators, were also used when necessary and feasible. Functional outcome was analyzed based on Glasgow Outcome Scale Extended (GOSE) through telephone interviews conducted by a research nurse one-year post ictus [23].

### Blood samples

CRP concentrations (reference interval < 5 mg/L) and WBC counts (reference interval 3.5–9 × 10^9^/L) were included in the standard blood testing protocol of our unit. CRP was analyzed in blood plasma samples by automated turbidimetric methodology. WBC counts were measured in whole blood samples by automated flow cytometric methodology. All analyses were performed in the Swedac accredited clinical chemistry laboratory of our hospital. Samples were collected on admission as well as daily during the hospital stay. In case of more than one test on the same day the mean values were used as the daily value.

### Statistics

Basic statistics included calculations of means and 95% confidence intervals (CI) as well as medians and 25–75% percentiles (interquartile range; IQR) for CRP and WBC for each day. Day 1 was considered to reflect Early Brain Injury and Day 4 the time point when from the clinical experience many complications occur; therefore, Day 1 and Day 4 values were further analyzed. Distribution of CRP and WBC values was tested visually with histograms and mathematically with Shapiro–Wilk and Kolmogorov-Smirnoff tests and the data were not normally distributed. Therefore, nonparametric methods were used for statistical analyses.

**Fig. 1:**
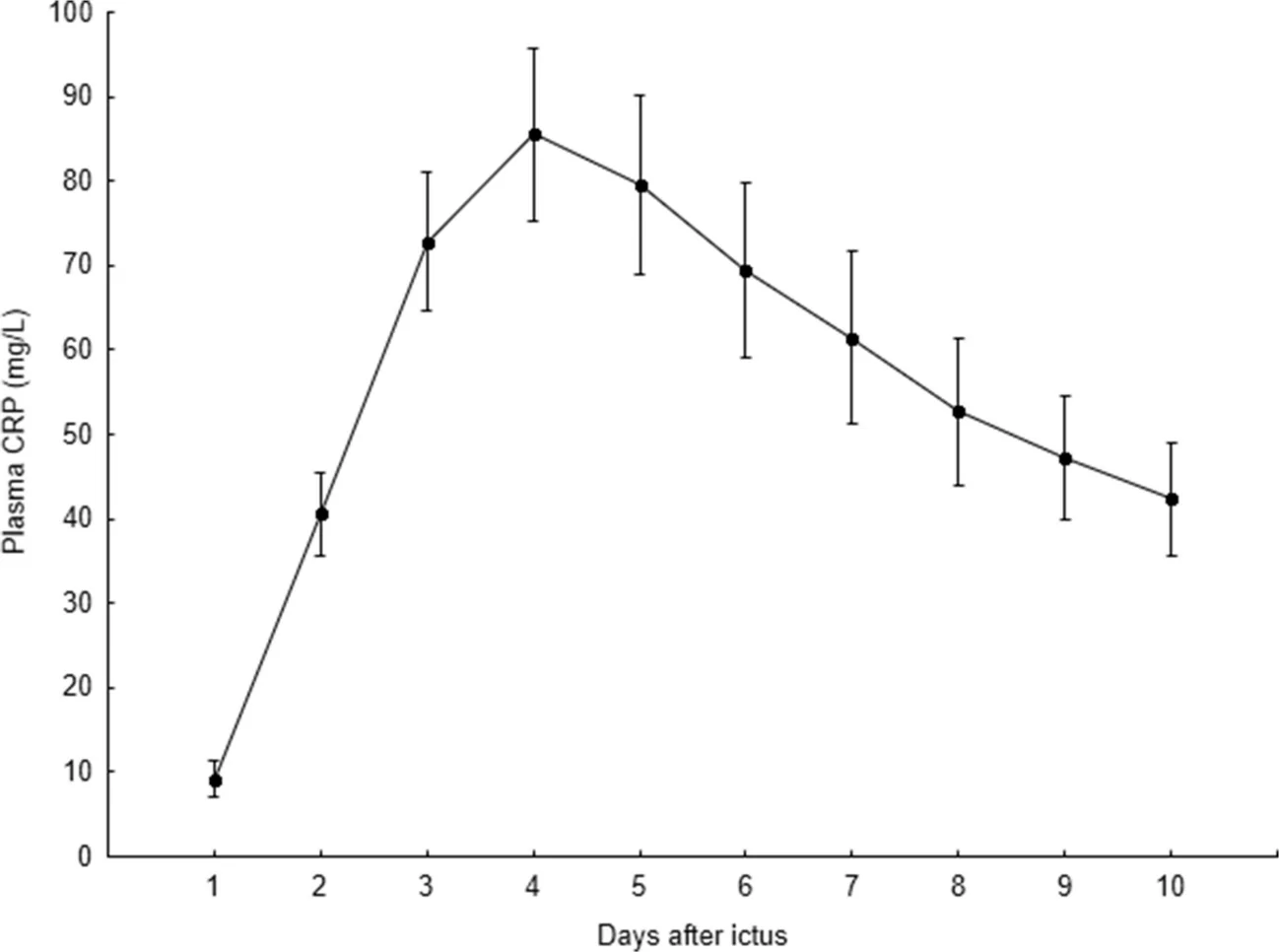
Temporal pattern of plasma CRP (means ± 0.95 CI) for Days 1–10 after SAH. Figure shows that mean values were slightly above normal range on Day 1 after ictus and then increased to a maximum on Day 4 after ictus

**Fig. 2:**
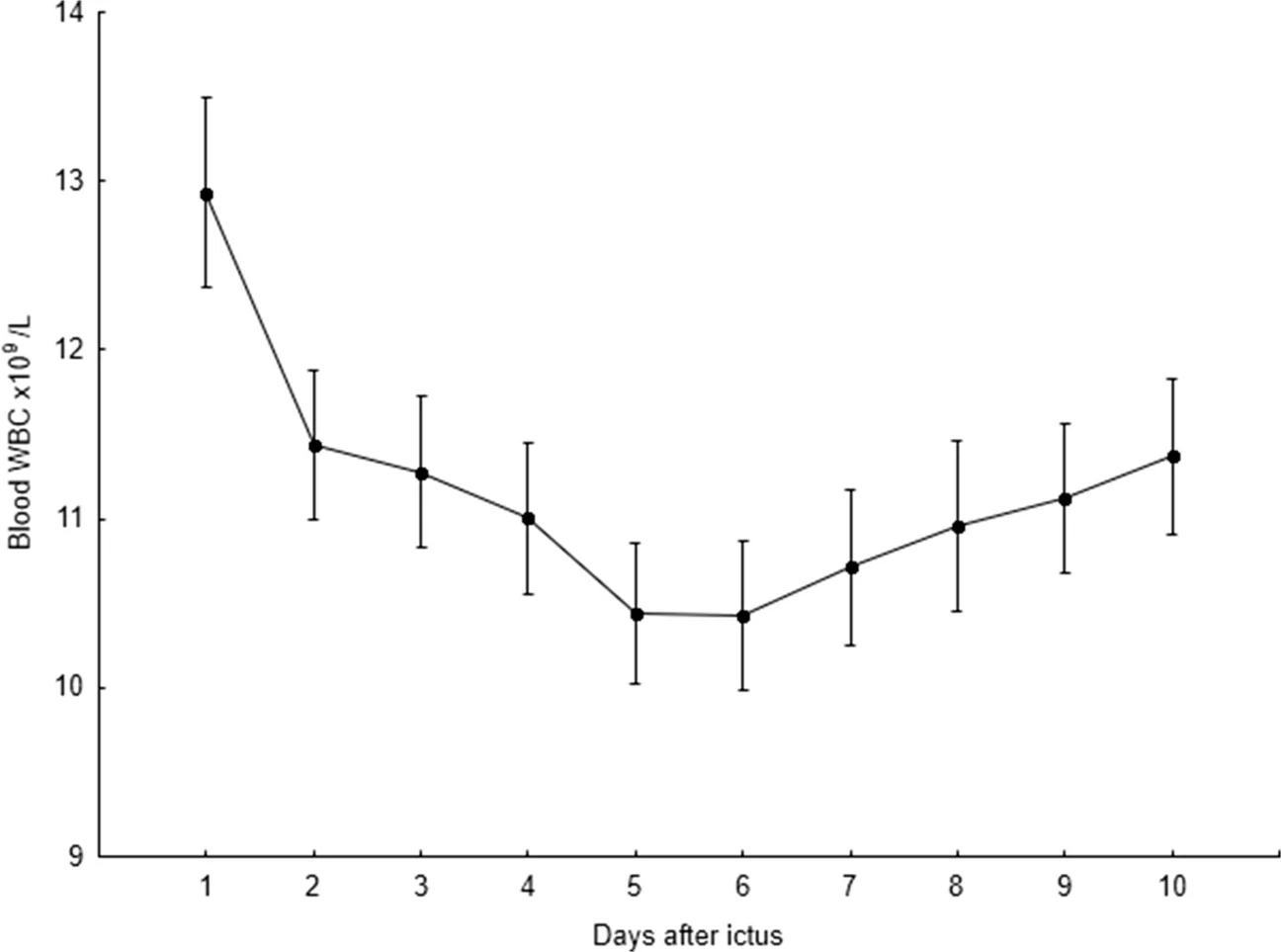
Temporal pattern of blood White Blood Cells (means ± 0.95 CI) for Days 1–10 after SAH. Figure shows that on the first day after ictus, the mean values were increased above normal range and showed the highest values during the monitoring period. Values then decreased towards normal levels on Days 5–6 after ictus, followed by a second peak

To evaluate the temporal patterns of CRP and WBC between Day 1 and Day 10, means and 95% CI for each parameter and day were plotted on graphs (Figs. 1 and 2). Friedman’s ANOVA was used on all samples to assess the repeated measures. To determine how Day 1 and Day 4 values were affected by demographics, Early Brain Injury and complications during the acute phase of the disease, univariate and multivariate analyses were performed.

Mann Whitney U test was used for univariate analysis. The patients were dichotomized in categorical groups female (yes; no); identifiable bleeding source (yes vs no; in essence, aneurysmal vs non-aneurysmal SAH); Fisher 3–4 (as opposed to Fisher 1–2); RLS 4–8 (as opposed to RLS 1–3; that is, unconscious vs conscious on admission); occurrence of EVD (yes; no); infection (yes; no); and DIND (yes; no). Day 1 samples were obtained on admission before insertion of EVD and also before events such as infections and DIND that may occur after some days. Therefore, EVD insertion, infections and DIND were not presumed to affect Day 1 results, so they were only included in the Day 4 values analysis.

Multivariate analysis was performed with general linear/nonlinear models of best subsets of variables according to Akaike’s Information Criterion (AIC) in order to identify the models best predicting increased CRP and WBC values on Day 1 and Day 4 [19]. For Day 1 values categorical factors included in the model were female sex, identifiable bleeding source, Fisher 3–4, and unconscious at admission (RLS 4–8). Age was used as continuous predictor. For Day 4 values the categorical factors also included events during the acute phase, i.e. occurrence of EVD, infection, and DIND in addition to the above-named variables. Models were performed with gamma distribution as best fitting model. The statistical software evaluated models for every possible subset of variables and returned the top 200 ranked by AIC. The relative likelihood of a model compared to the best (minimum AIC) model was estimated using the formula exp((AIC_min_ – AIC_model_)/2) [47]. For example, a relative likelihood of 0.5 means that the model is half as likely to be the best model as is the top ranked model.

All statistical analyses were performed with Statistica software (Stat Soft, Inc., Tulsa, OK, USA). Results were considered significant at *p*-value of < 0.05.

## Results

Table 1 summarizes the demographic data of the patient cohort. Totally 552 patients were included. There were 209 male and 343 female patients, and the mean age was 57 years. For each day a different number of patients had blood samples taken, varying between 304 and 444. The ANOVA was significant for both CRP and WBC (*p* < 0.0001). Tables 2 and 3 show the results from univariate analysis for CRP and WBC. In the univariate analysis, stronger inflammatory response as expressed by significantly higher CRP and WBC counts on Day 1 and Day 4 was generally noticed in more severe bleeding and complications. There was no difference between males and females.

**Table 1:** Demographic data of the patient cohort

N	552							
Sex	**Male**				**Female**			
	209				343			
Mean age	56.8							
Angiography result (Bleeding source)	**Identified**				**Not found**			
	398				154			
Admission Fisher score (Median 3)	**1**		**2**		**3**		**4**	
	40		133		238		141	
Admission RLS score (Median 2)	**1–3**				**4–8**			
	472				80			
EVD under observation period	**Yes**				**No**			
	245				307			
Infections (Isolated or multiple)	**Yes**				**No**			
	184				368			
Occurrence of DIND	**Yes**				**No**			
	94				458			
GOSE score (unknown = 29)	**1**	**2**	**3**	**4**	**5**	**6**	**7**	**8**
	74	0	67	70	25	134	125	29

Bleeding source included one or multiple aneurysms (*n* = 383); arteriovenous malformations (*n* = 5), arteriovenous fistulas (*n* = 6) and miscellaneous such as cavernous malformations, Moya Moya disease and arterial dissections (*n* = 4). *RLS* Reaction Level Scale, *EVD* external Ventricular Drainage, *DIND* Delayed Ischemic Neurologic Deficit, *GOSE* Glasgow Outcome Scale Extended

**Table 2:** Results from univariate analysis for CRP Day 1 and Day 4 values

		No	Yes	*p*-value
Female	Day 1	3.1 (1.3–7.6)	3.4 (1.3–8.8)	0.6271
	Day 4	26 (6.2–108)	38 (7.2–107)	0.7395
Identified bleeding source	Day 1	1.7 (0.9–4)	4.2 (1.7–9.3)	< 0.0001
	Day 4	8.8 (2.6–19)	52.5 (9.4–114.5)	< 0.0001
Fisher 3–4	Day 1	1.9 (0.9–5.2)	3.9 (1.6–9.1)	< 0.0001
	Day 4	7.6 (2.6–35)	54 (11–116)	< 0.0001
RLS 4–8	Day 1	2.8 (1.1–6.9)	8.4 (3.4–16.5)	< 0.0001
	Day 4	19.5 (5.3–87)	113 (62–159)	< 0.0001
EVD	Day 1	NA	NA	NA
	Day 4	7.3 (2.6–18)	88 (43–140)	< 0.0001
Infection	Day 1	NA	NA	NA
	Day 4	10.5 (3–45)	87 (45–140)	< 0.0001
DIND	Day 1	NA	NA	NA
	Day 4	19 (4.9–91)	74 (35–133)	< 0.0001

CRP Day 1 and Day 4 median values and interquartile range (mg/L) in dichotomized groups of patients. Results were considered significant when *p* < 0.05. Day 1 samples were collected on admission before EVD insertion so occurrence of EVD as well as infections and DIND that occur later were not presumed to affect Day 1 results and were only compared for Day 4 values. *CRP* C-reactive protein, *RLS* Reaction Level Scale, *EVD* External Ventricular Drainage, *DIND* Delayed Ischemic Neurologic Deficit, *NA* not applicable

**Table 3:** Results from univariate analysis for WBC Day 1 and Day 4 values

		No	Yes	*p*-value
Female	Day 1	11.1 (9–14)	11.6 (9.5–14.4)	0.4172
	Day 4	10.5 (8.5–12.5)	10.4 (8.5–12.5)	0.9006
Identified bleeding source	Day 1	10.1 (8.1–12.4)	11.9 (9.8–15)	< 0.0001
	Day 4	9.9 (7.9–12.1)	10.6 (8.8–12.7)	0̄.0497
Fisher 3–4	Day 1	9.5 (7.8–11.8)	12 (10–15)	< 0.0001
	Day 4	9.1 (7.7–12)	10.7 (9–12.7)	0.0008
RLS 4–8	Day 1	11.2 (9.1–13.8)	13.3 (10.1–15)	0.0019
	Day 4	10.4 (8.5–12.5)	11.1 (8.1–12.8)	0.6270
EVD	Day 1	NA	NA	NA
	Day 4	9.7 (8–12.2)	10.8 (9.1–12.8)	0.0011
Infection	Day 1	NA	NA	NA
	Day 4	9.8 (8.1–12.1)	11.1 (9.1–13)	0.0007
DIND	Day 1	NA	NA	NA
	Day 4	10.1 (8.2–12.3)	11.3 (9.2–13.4)	0.0029

WBC Day 1 and Day 4 median values and interquartile range (× 10^9^/L) in dichotomized groups of patients. Results were considered significant when *p* < 0.05. Day 1 samples were collected on admission before EVD insertion so occurrence of EVD as well as infections and DIND that occur later were not presumed to affect Day 1 results and were only compared for Day 4 values. *WBC* White Blood Cells, *RLS* Reaction Level Scale, *EVD* External Ventricular Drainage, *DIND* Delayed Ischemic Neurologic deficit, *NA* not applicable

Multivariate analysis was done including five variables for Day 1 and eight variables for Day 4, as described in the statistics section above. The results show the three models that best predicted higher concentrations of CRP and WBC on Day 1 and Day 4 (Table 4). CRP was slightly increased on Day 1 (Fig. 1) and higher levels were predicted by models including increasing age, female sex, and markers for a more severe hemorrhage. WBC were increased on Day 1 (Fig. 2) and higher levels were predicted by models including increasing age and markers for a more severe hemorrhage (Table 4). CRP increased markedly during the following days with a peak on Day 4 (Fig. 1). This value was best predicted by models including female sex, a more severe hemorrhage, EVD, various infections, and increasing age (Table 4). WBC displayed a different pattern with decreasing levels after admission (Fig. 2). Higher levels of WBC on Day 4 were best predicted by models including more blood in the hemorrhage (i.e., Fisher 3–4), presence of infection, and presence of DIND.

**Table 4:** Results from multivariate analysis

	*P*-value	AIC	Relative likelihood
**CRP Day 1**			
Female; bleeding source; RLS 4–8; age	2.39 10^–13^	2497.001	1
Bleeding source; RLS 4–8; age	2.87 10^–13^	2498.696	0.428
Female; bleeding source; Fisher 3–4; RLS 4–8; age	1.04 10^–12^	2499.001	0.367
**WBC Day 1**			
Bleeding source; Fisher 3–4; RLS 4–8; age	< 1 10^–17^	2282.445	1
Female; bleeding source; Fisher 3–4; RLS 4–8; age	< 1 10^–17^	2283.672	0.541
Bleeding source; Fisher 3–4; age	< 1 10^–17^	2284.364	0.383
**CRP Day 4**			
Female; bleeding source; RLS 4–8; EVD; Infection; age	< 1 10^–17^	4055.061	1
Female; bleeding source; Fisher 3–4; RLS 4–8; EVD; Infection; age	< 1 10^–17^	4056.054	0.608
Female; bleeding source; RLS 4–8; EVD; Infection	< 1 10^–17^	4056.203	0.564
**WBC Day 4**			
Fisher 3–4; infection; DIND	0.000035	2143.131	1
Fisher 3–4; infection; DIND; age	0.000063	2143.881	0.687
Bleeding source; Fisher 3–4; infection; DIND	0.000083	2144.477	0.511

Models best predicting higher CRP and WBC values on Day 1 and Day 4 after SAH. Models were performed with gamma distribution as best fitting model. Categorical variables for Day 1 (both CRP and WBC) included female sex, bleeding source, Fisher 3–4 and RLS 4–8. Age was used as continuous predictor. EVD insertion, Infection and DIND were only analyzed for Day 4 since they occurred after Day 1 samples were obtained and they were not presumed to affect Day 1 results. Columns are showing combinations of predicting variables, statistical significance, Akaike’s Information Criterion value (minimum = best possible) and relative likelihood of subsequent models compared to best possible model, calculated by the formula exp((AIC_min_ – AIC_mod_)/2). *RLS* reaction level scale, *EVD* external ventricular drainage, *DIND* delayed ischemic neurologic deficit

**Fig. 3:**
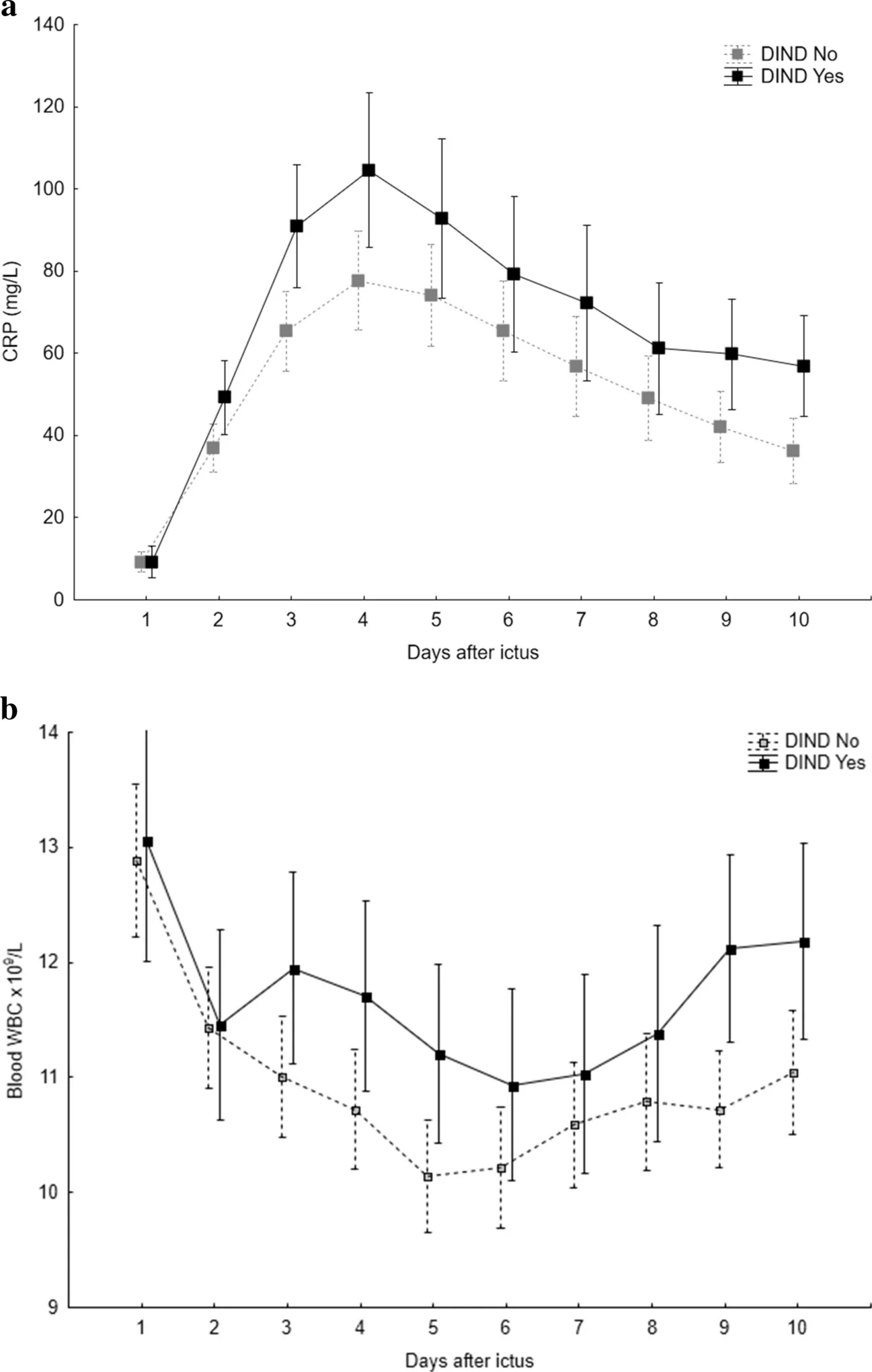
**a** Temporal profiles of CRP (means ± 0.95 CI) in the subgroups DIND vs NO DIND. Figure shows similar trends between the two groups that are also similar to the CRP trend for all patients, as shown in Fig. 1. **b** Temporal profiles for blood WBC (means ± 0.95 CI) in the subgroups DIND vs no DIND. Figure shows different trends over time and that higher WBC counts for patients with DIND were present already on Day 3

**Fig. 4:**
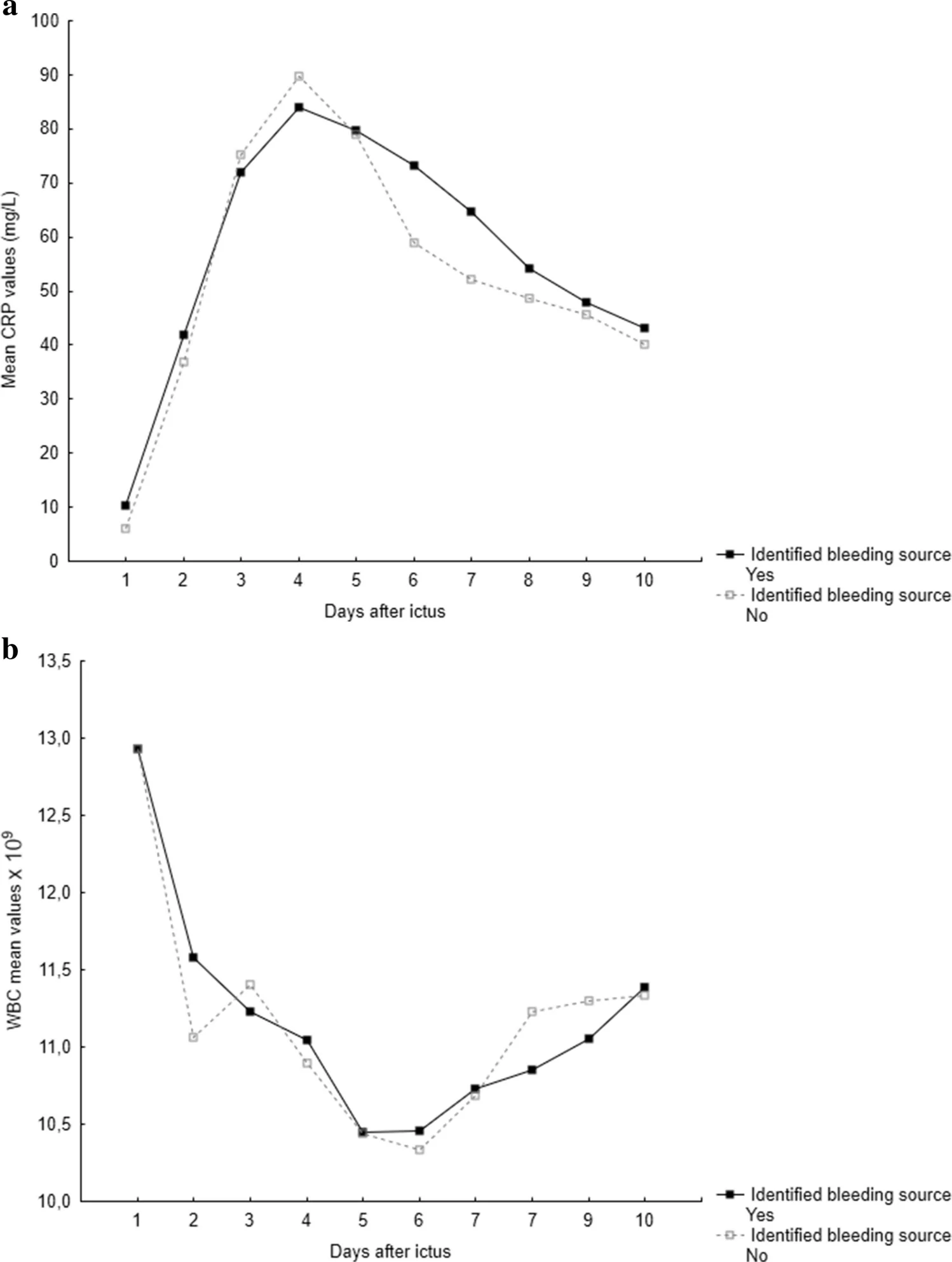
**a** and **b** Temporal profiles of mean CRP (**a**) and WBC (**b**) values in subgroups “Identified bleeding source” vs “No bleeding source” (in essence, aneurysmal vs non-aneurysmal SAH) illustrating similar inflammatory trends

The temporal profiles for CRP and WBC in the subgroups DIND vs no DIND were plotted in Fig. 3a and b. Regarding CRP, a difference in the levels between patients with and without DIND was illustrated starting day 2, but essentially no difference in the trends over time (Fig. 3a). Figure 3b on the other hand showed different temporal trends between the subgroups and that higher WBC counts for patients with DIND were present already on Day 3. The temporal profiles for WBC and CRP were further plotted for the subgroups with or without identified bleeding source. The figures showed no obvious difference in the levels or trends over time for either parameter between the groups (Fig. 4a and b).

## Discussion

In the present study two widely used inflammatory markers such as CRP and WBC were measured in blood of SAH patients in a large, consecutive cohort representing the whole spectrum of the disease. Blood was preferred over other samples such as CSF since blood tests were part of the standard daily work up throughout the whole observation period in all patients (not requiring the presence of EVD that usually is needed in more severe bleedings, leading to some selection bias), thus providing a large number of observations and more robust statistical results. CRP and WBC were chosen despite being non-specific since they can be easily analyzed in blood and are available essentially everywhere allowing for serial monitoring of the inflammatory response. Fever as a clinical marker of inflammation was also contemplated for analysis but was opted out due to inconsistency in recording between patients and aggressive antipyretic treatment directly when fever was noticed in accordance with the clinic’s SAH protocol.

The most striking findings of the study come from the results of multivariate analysis for Day 4 values. According to our results, female patients with aneurysms, unconscious on admission, with EVD, infection, and increasing age were the most likely combination of having higher CRP values on Day 4 (Table 4). Interestingly, DIND was not among the parameters included in the three best predicting models, even though one might expect an association of increased CRP and occurrence of DIND, especially looking on the CRP trajectory of Fig. 1 where the CRP peak coincides temporally with the occurrence of DIND that is observed in the clinical praxis. A possible explanation may be that CRP is a highly nonspecific marker of inflammation that is affected by many stimuli and not particularly from the processes that lead to DIND. Inconsistent results on this matter are found in the literature with some studies reporting correlations between levels of CRP and DCI/DIND and others failing to show such relationships [8, 26, 31].

On the contrary, DIND together with Fisher 3–4 and infections were included in the best predicting model of higher WBC counts on Day 4 (Table 4). Previous studies have also pointed to a correlation between WBC counts and DCI/DIND [11, 30, 31]. This association is not obvious when one looks on the temporal trajectory of WBC counts in Fig. 2 since the lowest values are observed on Days 5 and 6 when DIND usually occurs. However, a closer look on the specific trajectories of each group (i.e., DIND vs no DIND) reveals a sharp increase of WBC counts on Day 3 for patients with DIND (Fig. 3). Kasius et al. reported a similar increase of WBC counts (alongside with platelets) in serum shortly before onset of DCI/DIND unrelated to treatment and Provencio et al. reported increased neutrophil percentage in CSF to be an independent predictor of vasospasm defined by transcranial Doppler and angiographies [25, 34]. Other features apart from total leukocyte count such as the rate of change or a peak value of > 15 × 10^9^/L have also been associated with DIND [2]. All these results highlight a possible important role of WBC in the pathogenesis of this potentially detrimental complication, although the lack of specificity makes WBC less suitable to be used as a biomarker.

The temporal patterns for CRP and WBC were noticed to differ substantially (Figs. 1 and 2). While CRP increased steeply until Day 4, followed by a gradual decrease until Day 10, the highest levels of WBC were noticed early after ictus, i.e., on Day 1. This early peak of WBC could reflect their pivotal role in the brain pathophysiology of SAH with early activation and proliferation, migration to the bleeding site, phagocytosis and release of inflammatory cytokines to further mediate the inflammatory response [13, 16, 18]. CRP on the other hand may reflect the temporal pattern of the inflammatory response post SAH in a way closer to the one observed in the clinical practice with a peak around Day 4 and gradual decline towards Day 10. Similar CRP and WBC trajectories have been reported by Hollig et al. [20].

Patients with more severe bleeding (i.e., with aneurysms, Fisher 3–4, and RLS 4–8) showed higher Day 1 CRP and WBC values and those with complications such as infections and DIND had significantly higher Day 4 values as well, results that support a clear association of inflammation with disease severity. This is in accordance with findings from previous studies, including even the CSF compartment [9, 15, 37]. However, such results from univariate analyses should be interpreted cautiously since the inflammatory response post SAH is a very complex phenomenon and cannot be adequately described only by studying individual variables. This problem may be partially overcome by multivariate analysis that considers the complex interplay between different variables. For example, almost all studied parameters were included in the models best predicting higher Day 1 values for both CRP and WBC as seen in Table 4 (except for Fisher 3–4 for CRP and female sex for WBC).

We included a number of patients where no bleeding source was identified despite repetitive angiographic examinations (*n* = 154; 27%). Spontaneous non-aneurysmal SAH (NA-SAH) is generally considered to be a benign entity, distinctly separate from aneurysmal SAH in terms of clinical course, need of therapeutic interventions and functional outcome [36, 45]. However, a closer look on the demographics of the subgroup of NA-SAH patients in our cohort reveals occurrence of DIND (*n* = 6/154; 3,8%), need of EVD insertion (*n* = 20/154; 13%) and eventually unfavorable outcome (*n* = 19/154; 12,3%) in a substantial number of patients (Table 5). Similar numbers are reported in other studies too and indicate that the condition is not that harmless and should not be neglected [33, 48]. We chose to analyze NA-SAH patients together with patients with aneurysms or other bleeding sources, since we believe that the pathophysiological mechanisms of inflammation that underlie both conditions are common and are elicited by the bleeding itself. This notion of comparable inflammatory responses becomes clear when one looks at the similar temporal trajectories of CRP and WBC mean values in the groups “Identifiable bleeding source” vs “No bleeding source”, as presented in Fig. 4a and b.

**Table 5:** Demographics of non-aneurysmal SAH cohort

N	154			
Sex	Male		Female	
	82		72	
Mean age	54.9			
Admission Fisher score (Median 3)	1	2	3	4
	23	57	66	8
Admission RLS score (Median 2)	1–3		4–8	
	150		4	
EVD under observation period	Yes		No	
	20		134	
Infections (Isolated or multiple)	Yes		No	
	15		139	
Occurrence of DIND	Yes		No	
	6		148	
Outcome (unknown = 13)	Favorable (GOSE 5–8)		Unfavorable (GOSE 1–4)	
	122		19	

*RLS* Reaction Level Scale, *EVD* External Ventricular Drainage, *DIND* Delayed Ischemic Neurologic Deficit, *GOSE* Glasgow Outcome Scale Extended

### Limitations

The study is a single-center study with retrospective design. The absence of similar analyses in other compartments such as intrathecally, although a conscious decision as described earlier, still can be considered a limitation. Events such as mechanical ventilation in the ICU or surgical clipping of the aneurysm as opposed to endovascular coiling may affect CRP and WBC values and the lack of specific analyses about these parameters is a limitation. Best models based on AIC was preferred in the multivariate analysis to odds ratio that is frequently used together with logistic regression since the latter may overestimate the value of a single variable while the former represents more reliably the clinical setting with many confounding variables. Finally, it is important to note that the current study is not predictive, since no ROC analysis or other predictive performance measures were performed. It is rather an explorative study of the early phase of SAH inflammation, describing associations between CRP/WBC values and common SAH complications. The term “predict” that is used when describing the results of the multivariate analysis above is used in its statistical sense and should not be interpreted as implying clinical prediction.

## Conclusions

More severe initial bleeding and occurrence of complications in the early phase of SAH are generating a more intense inflammatory response in terms of elevated CRP and WBC concentrations. CRP trajectory from Day 1 to Day 10 may be considered to better resemble the clinical course of the disease, probably due to CRP’s nonspecific nature that is affected by many different inflammatory stimuli apart from the bleeding itself, such as infections, surgical trauma etc. Leukocytes peak early which is in line with their established role in the orchestration of inflammatory response early post SAH. Occurrence of DIND may be associated with an increase in WBC around Day 4 which can possibly be used for early detection of this important complication, although cautiously and in combination with other clinical and laboratory markers due to its non-specificity.

## Data Availability

Generated research data can be available upon reasonable request.
